# An Evolution of the Standards to Enhance Gerontological and Geriatric Education

**DOI:** 10.1093/geroni/igaa057.1813

**Published:** 2020-12-16

**Authors:** Tamar Shovali, Karen Kopera-Frye, Joann Montepare

**Affiliations:** 1 Eckerd College, St Petersburg, Florida, United States; 2 New Mexico State University, Las Cruces, New Mexico, United States; 3 Lasell University, Newton, Massachusetts, United States

## Abstract

This presentation will focus on AGHE’s evolving discussions on gerontological and geriatric programming. The earliest discussion resulted in development of the first edition of the standards and guidelines for programs in 1989. In 2014, the AGHE Academic Program Development Committee (APDC) created new core competencies for gerontological education. Currently, members from both the AGHE APDC and Advancement Subcommittees are collaborating on revising the AGHE Standards and Guidelines (6th edition, 2015) to align with competencies. This presentation will discuss the new and improved version (draft available in November 2020). First, presenters will review points regarding how this update will aid in Accreditation for Gerontology Education Council and Program of Merit self-study program reviews. Secondly, the presenters will explain how the revision will increase gerontological education program integrity. Lastly, the presenters will explain how this update will be consistent with the trend across institutions utilizing these competencies in gerontological and geriatric education. Part of a symposium sponsored by the Geriatric Education Interest Group.

